# Traumatic Burst Fracture with Spinal Channel Involvement Augmentation with Bioactive Strontium-Hydroxyapatite Cement

**DOI:** 10.1155/2013/613149

**Published:** 2013-08-01

**Authors:** S. Masala, E. Calabria, G. Nano, R. Iundusi, L. Greco, R. Di Trapano, U. Tarantino, G. Simonetti

**Affiliations:** Department of Diagnostic Imaging, Molecular Imaging, Interventional Radiology, and Radiation Therapy, University Hospital Tor Vergata, Viale Oxford 81, 00133 Rome, Italy

## Abstract

In November 2011 a 75-year-old man was admitted to our emergency department with a low back pain caused by a traumatic L1 vertebral collapse with backward projection of posterior wall superior third. The indication for neurosurgical instrumentation was placed, although he refused the treatment. Hence he was treated conservatively without a significant improvement up to January 2012 when, still refusing surgery, he accepted to undergo percutaneous vertebroplasty with a novel bioactive injectable strontium-hydroxyapatite cement. Vertebroplasty was performed without complications. A CT scan, performed the day after the procedure, ruled out extravertebral cement leakage. Pain improvement was significant (preprocedure VAS 10, one-week VAS 4) with a gradual decrease up to three months when it stabilized at 2. CT examination after 1 year showed a good cement osseointegration with osteophytic spurs bridging the superior endplate of the level involved to the inferior one of the level above. The new bone ingrowing property of the strontium-hydroxyapatite containing cement permits to extend the treatment indication also to unstable collapses in which the risk of pseudoarthrosis is very high. In this reported case we evaluated the potential role of percutaneous vertebroplasty in traumatic burst fracture with spinal channel involvement.

## 1. Introduction

Acute vertebral compression fractures are common and can occur secondary to osteoporosis, trauma, neoplasm, metastasis, or myeloma [1].

Different approaches for the management of painful VCFs are currently available, but the treatment depends on the neurological status of the patient, features of the fracture, and its pathogenesis. Numerous improvements have been made in the last years, due to the introduction in clinical practice of minimally invasive techniques such as percutaneous vertebroplasty (PVP) and kyphoplasty (PKP).

Both are reported to ensure a faster consolidation of fracture with a significative improvement of pain in comparison with the conservative treatment [2, 3]. 

PVP is considered a very safe and effective procedure in treating painful primary or secondary osteoporotic refractory to medical therapy, compression fractures caused by osteonecrosis [4, 5], or neoplasm [6–8].

PVP was first reported in the literature in 1987 by Gailbert et al., for the treatment of a cervical vertebral hemangioma [9].

In the following years, evolution of techniques, evolution of cement, and expansions of indications have resulted in several useful vertebral augmentation procedures [10].

PVP on patients with traumatic fractures might increase the rate of cement posterior extravasation with possible spinal injuries or damage to posterior and anterior longitudinal ligaments [11]. 

For these reasons, recently PKP is used in clinical practice for the treatment of traumatic vertebral fracture [12, 13].

Type A1.2 and 3, A2.2, and A3.1 are treated with kyphoplasty, which warrants a more controlled injection of the bone cement, allowing a more precise reconstruction and a stabilization of vertebral fractured body [14].

The conventional vertebral cement augmentation technique balloon kyphoplasty has become a popular tool to address painful thoracic and lumbar compression fractures [15, 16].

It showed pain reduction and lower complication rates compared to standard PVP [17, 18]. 

A 75-year-old man with L1 A1.3 traumatic fracture underwent PVP with a novel bioactive strontium-hydroxyapatite bone cement. Vertebral collapse characteristics had put indication to surgical instrumentation, but the patient refused. He received a conservative treatment, but the pain did not resolve. Despite receiving this treatment, the pain did not subside. Therefore he accepted to undergo PVP. 

Polymethylmethacrylate (PMMA) cement embeds some disadvantages among which the leading one, concerning this case, is high stiffness and consequent foreign body reaction at bone-PMMA interfaces with risk of pseudoarthrosis and instability. To avoid such complications, which in case of vertebral channel involvement by fragmented posterior wall could be very harmful, we opted for a strontium-hydroxyapatite cement with lower stiffness and bone ingrowing properties. 

After the treatment the pain decreased almost immediately going from preprocedure 10 VAS points to stabilization on 2 after three months.

## 2. Case Report

In November 2011 a 75-year-old man was admitted to our emergency department, after a motor vehicle accident, with low back pain that was improved by lying in a supine position and exacerbated by any attempt to elevate the head of the stretcher. 

On physical examination the pain could be reproduced on palpation or percussion over the spinous process of the affected level. At plain X-ray L1 vertebral collapse with minor loss of vertebral body height and irregular posterior wall outline was evident. Noncontrast lumbar spine computed tomography (CT) (Lightspeed VCT, GE Healthcare Medical Systems, Milwaukee, WI, USA) performed straight afterwards ruled out an A1.3 collapse with vertebral channel involvement by the superior third of the posterior wall.

The patient was not osteoporotic and in good clinical conditions, and he had no history of multiple myeloma or long-term steroid use; thus, indication to neurosurgical instrumentation was placed, but the patient refused the intervention. the option of total bed rest for several months without any mobilization was considered unacceptable by both the patient and his family. So he was discharged home with bracing and painkillers.

In January 2012 the patient presented at our outpatient ward in poor clinical conditions because of pain-induced daily life activities compromise. Although no benefits derived from both painkillers and bracing, he continued to refuse surgery. 

Therefore we proposed him percutaneous vertebral augmentation with a novel injectable bioactive strontium-hydroxyapatite (Sr-HA) bone cement (Osteo G-ASPINE, San Francisco, CA, USA) with the aim to stabilize the collapse and improve the clinical setting. The patient was informed about the possible complications due to the procedure, and a written informed consent was obtained.

**Figure 1:**
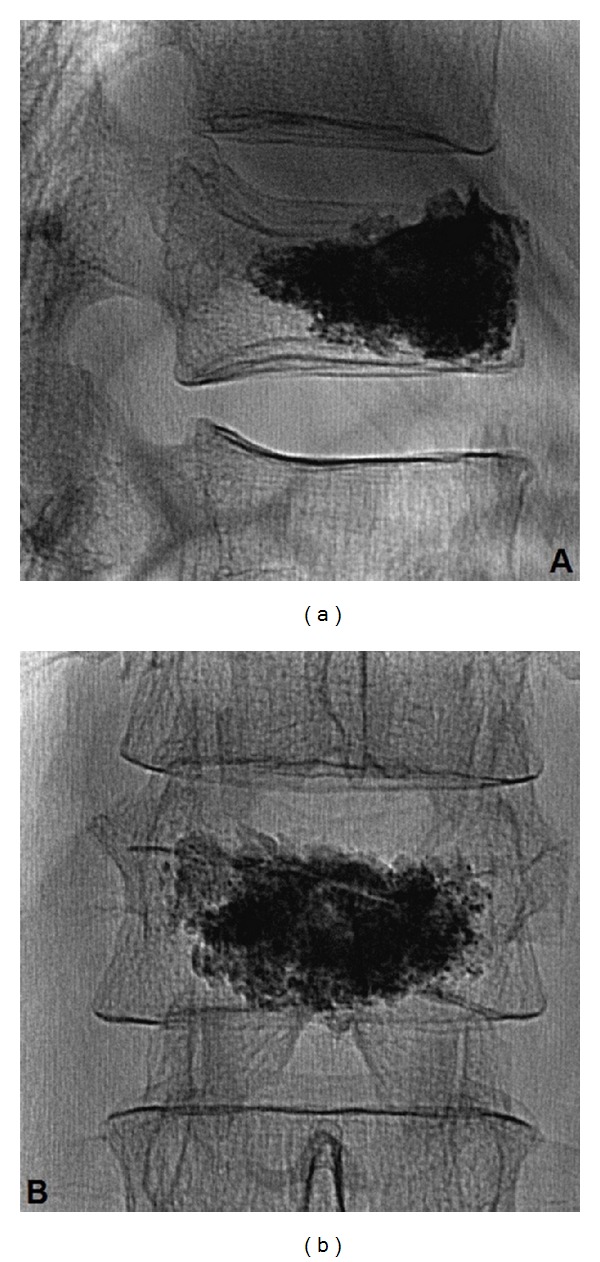
Lateral (a) and anteroposterior (b) end-procedure fluoroscopic views showing optimal cement spreading inside vertebral body; note the excellent opacity of the strontium-containing cement.

The patient was placed in the prone position on the radiological table with the spine extended. Continuous monitoring of pulse rate, oxygen saturation, and blood pressure was applied during the procedure. His back was prepared in the usual sterile fashion and 1% lidocaine local anesthesia administered up to the periosteum. A small skin incision was made to access the pedicles. The fracture level was observed, and fluoroscopically guided (Allura, Philips, Netherlands) unilateral transpedicular approach was performed, with an 13-gauge bone biopsy needle. The needle was introduced into L1 body through the left pedicle. The bioactive Sr-HA bone cement was injected inside the vertebral body with uniform distribution by using 4 mL syringes under fluoroscopic control (Figure 1). 

After the extraction of the needle the incision did not require suturing, and the patient was instructed to remain in bed in supine position for the following 4 hours. No intra-or postprocedure complications occurred. The duration of the whole procedure was about 32 minutes. Antibiotics were administered 1 day before and 4 days after the procedure. 

**Figure 2:**
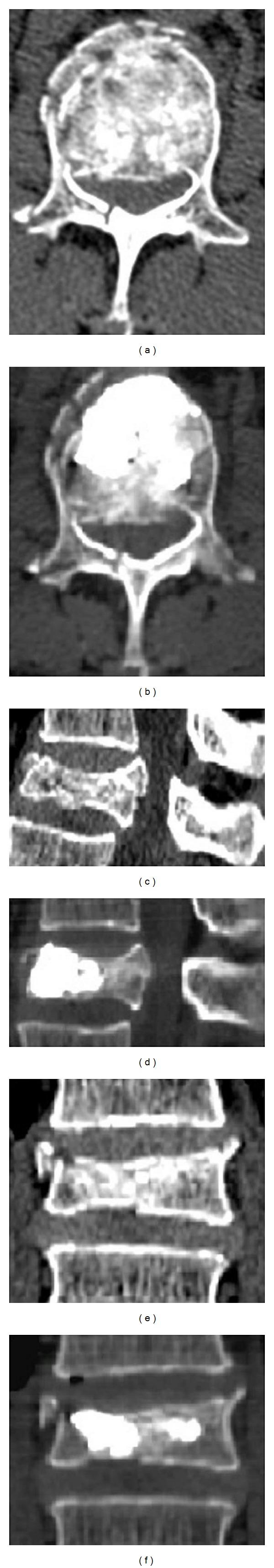
Pre-(left) and post-(right) procedure CT control in axial (top), sagittal (middle), and coronal (bottom) multiplanar reformations showing the good cement distribution throughout the anterior two-thirds of the collapsed vertebral body.

A CT scan performed the day after the procedure ruled did not show cement leakage (Figure 2, left column).

The patient was able to ambulate independently on first by procedure day and was discharged 2 days after the procedure.

Pain relief was rapid and significant immediately after the procedure, starting from 10 (on 11 point visual analog scale VAS) preprocedure to stabilization on 2 after three months; patient pain improvement and clinical setting have been followed up to now (March 2013) being stable on VAS 2 with normal daily life performance status (Table 1).

## 3. Discussion

Vertebral augmentation for the treatment of vertebral body fractures or for preventive stabilization of an osteoporotic vertebral body is effective [19]. 

PMMA is the most widely employed and up to now the most cost-effective bone cement for PVP; however there are some disadvantages. It lacks osseointegration because of its high mechanical stiffness and chemical surface characteristics that may lead to the formation of a soft fibrous layer at the bone-cement interface with the potential of pseudoarthrosis with following pain relapses.

The Sr-HA bone cement has a number of advantages over the commonly used PMMA, despite its higher cost. Osseointegration occurs with Sr-HA and does not with PMMA [20–22]. Osseointegration will likely result in better long-term outcome with reduced risk of loosening, and it also raises the possibility of using Sr-HA cement to augment traumatic vertebral body collapses in young patients [23]. PMMA cement evokes an inflammatory response and foreign body reaction in the surrounding bony tissues; this is likely due to the presence of the toxic methyl methacrylate [MMA] monomer. In the Sr-HA specimens, no foreign body reactions, no inflammations, and no necrosis were observed by radiographic or histologic analyses. This is because Bis-GMA (methacrylic acid and bisphenol), the leading Sr-HA framework of this new cement, is nontoxic because of the lack of monomer decaying [24]. Sr-HA is superior to PMMA also because of its lower setting temperature and bone-like stiffness leading to a more uniform load transfer over the adjacent vertebral bodies, so the risk of recollapses decreases, both at the same level and at adjacent levels [25].

Our patient presented backward projection of the posterior wall without neural impingement putting him in a borderline field between traditional surgical instrumentation and semiconservative treatment by minimally invasive vertebral augmentation. One of the main contraindications to vertebral augmentation is posterior wall involvement with bone fragments, particularly at levels above L1, because of the risk of vertebral channel cement backward leakage with potential spine cord impingement and the risk of pseudoarthrosis with pain relapse [26]. 

Sr-HA injectability is good as PMMA, so the operator must take extreme care when injecting cement inside collapses with posterior wall involvement. This issue is also valid for Sr-HA cement; care must be taken when injecting it into A1.3 type collapse. 

Sr-HA permitted not only the safe injection in a fractured vertebral body with a posterior cortical breach, but also a uniform distribution and incorporation into the fracture lines. Moreover its radiopacity allows radiographic visualization without the need of additional radiocontrast material [24].

Patient worsening clinical condition owing to pain resistant to 3 months of conservative treatment together with his refuse to traditional neurosurgical instrumentation set an alternative minimally invasive therapeutic option mandatory. Therefore, once the patient was informed on risks and benefits, vertebral augmentation with Sr-HA was accomplished without any intra- or postprocedural complication. No cement leakage was evident at CT examination the day after procedure (Figure 2, left column). CT examination at 1 year has shown good cement osseointegration with osteophytic spurs bridging the superior endplate of the level involved to the inferior one of the level above (Figure 2, right column).

From the first week after the procedure, the patient reported a significant reduction of pain and disability, with further improvement at 1 month and stabilization of the symptoms at 3 months. Thus this new cement represents a valid alternative to surgery in cases of vertebral burst fractures without neural structures involvement. 

Sr-Ha cement for vertebral augmentation has proven effective in both pain and clinical setting improvement and vertebral stabilization also in this case of L1 A1.3 traumatic vertebral collapse widening indications and also to some selected cases in which the first option should be traditional neurosurgical instrumentation.

## Figures and Tables

**Table 1:** VAS scores before and after 1 week and 3, 6 months by the procedure of PVP. Pain relief was rapid and significant immediately after the procedure, starting from 10 preprocedure to stabilization on 2 after three months and 1 year after procedure.

	VAS score
Before	10
1 week	3.5
3 months	2
1 year	2
